# Primary Testis Leiomyosarcoma: A Case Report

**DOI:** 10.30699/ijp.2021.524644.2586

**Published:** 2021-07-06

**Authors:** Elham Nazar, Zohre Shabanzadeh, Amir Ahmadi, Niloofar Rostami

**Affiliations:** 1 ***Department of Pathology, Tehran University of Medical sciences, Tehran, Iran***; 2 ***Department of Pathology, Fajr Hospital, Mariwan, Iran***; 3 ***Department of Urology, Fajr Hospital, Mariwan, Iran***

**Keywords:** leiomyosarcoma, testis, orchiectomy

## Abstract

Primary leiomyosarcoma in testis is an uncommon tumor with few cases reported. It generally develops after radiotherapy or long-term taking anabolic steroid medication. We report a 53-year-old male patient with primary testis leiomyosarcoma who presented with painless testicular enlargement without any known predisposing factors. Ultrasound revealed a large heterogeneous left testicular solid lesion. Alpha-fetoprotein (AFP) and beta-human chorionic gonadotrophin (beta-HCG) levels in serum were normal. Left radical orchidectomy following with histology assessment established a diagnosis of primary leiomyosarcoma of testis. No data of cancer metastasis was established. The patient didn’t receive any adjuvant therapy. There wasn’t any evidence of recurrence after 1 year follow-up. Leiomyosarcoma must be one of the differential diagnoses of seronegative tumors in testis. The motivation for this paper is the extreme infrequency of the situation and the differential diagnosis by all expansive inguinoscrotal tumors.

## Introduction

Malignant spindle cell neoplasms have low frequency and are commonly sarcomas. Leiomyosarcoma accounts for 5%–10% of sarcoma (1). It is a tumor originating from smooth muscle cells (2). Leiomyosarcoma in testis is an uncommon tumor which might happen after radiotherapy, chronic inflammation, or anabolic steroids use for long time. However, in absent of risk factors, it is seldom found (3). Here we report a patient with primary leiomyosarcoma in testis, which was thought to derive from normal testis organ include smooth muscle cells, such as blood vessels or seminiferous tubules’ contractile cells.

## Case Presentation

**Fig 1:**
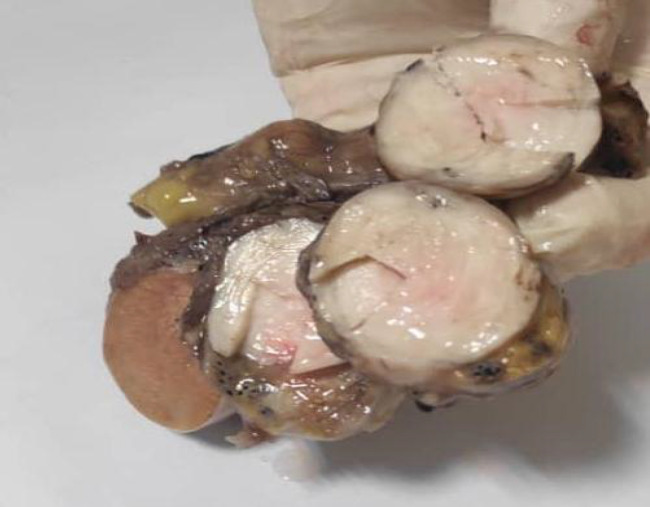
Sections show an intra-testicular whitish lesion with whorling pattern in upper pole measuring 4 × 3 × 3 cm

**Fig 2:**
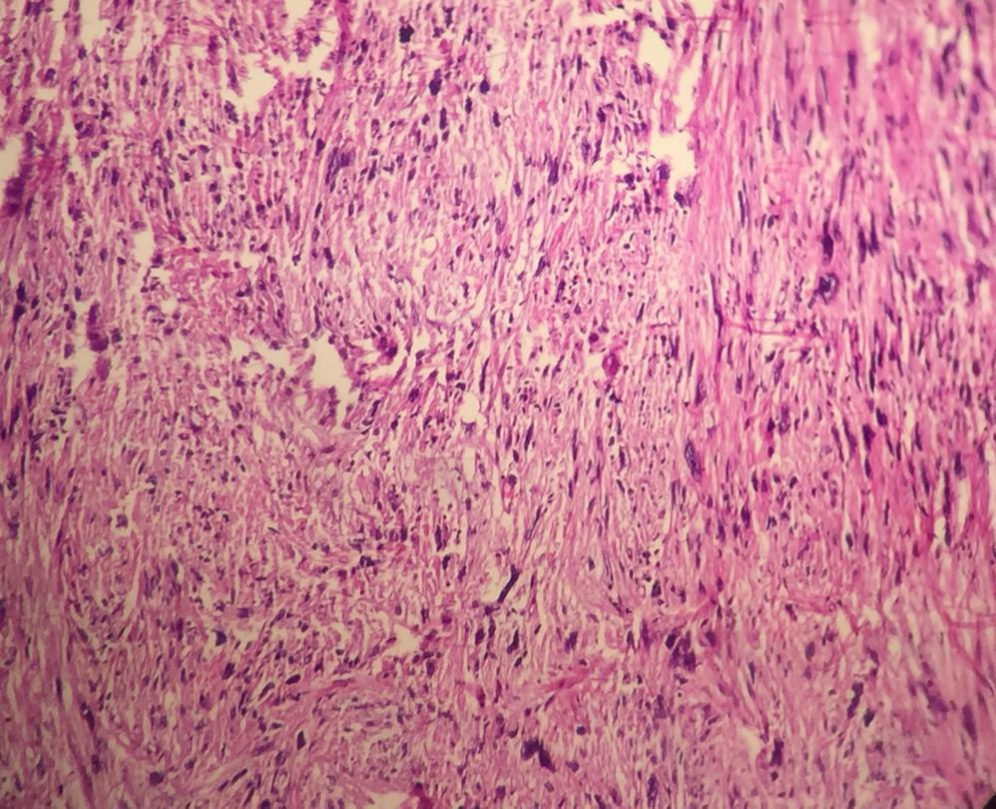
Histological examinations revealed a highly cellular neoplastic tissue composed of atypical cells with spindle to oval nuclei (× 40).

**Fig 3:**
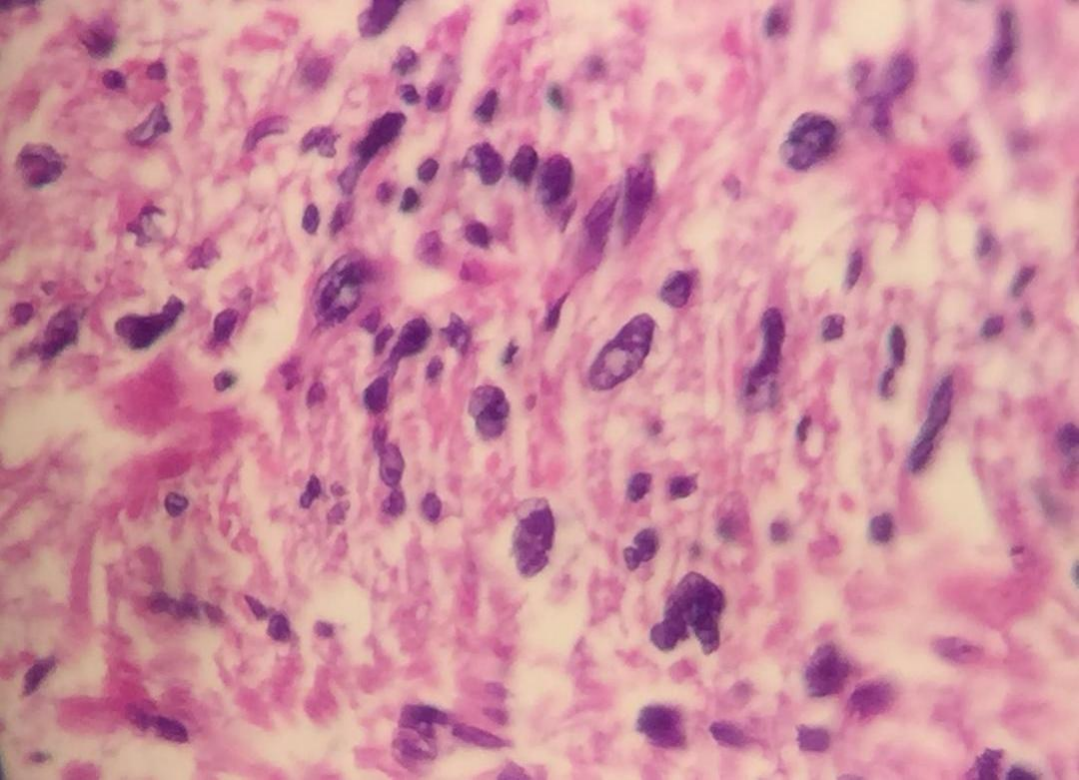
Histological examinations revealed atypical spindle to oval nuclei which showed marked pleomorphism (x400).

**Fig 4:**
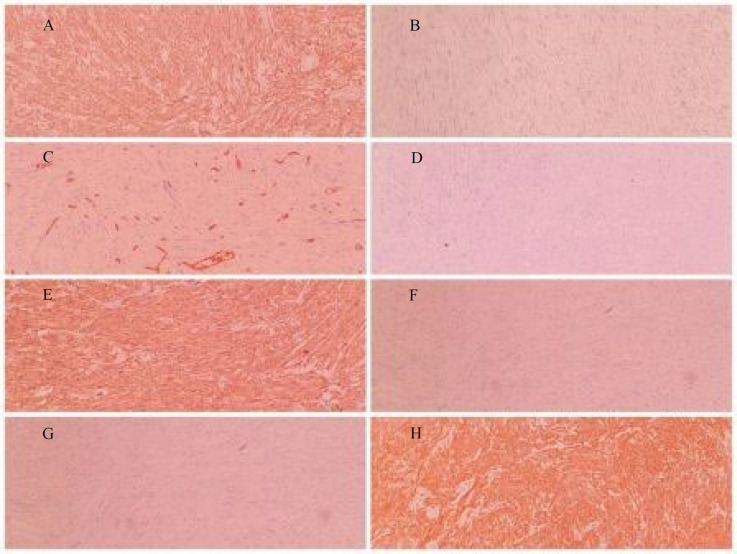
Immunohistochemical staining of primary leiomyosarcoma of the testis revealed (A) Positive SMA, (B) Negative CK, (C) Positive CD34 in blood vessels, (D) Negative C-Kit, (E) Positive H-Caldesmon, (F) Negative DOG-1, (G) Negative SOX-10, (H) positive Desmin

A 53-year-old male referred to the urology unit in Fajr hospital in Mariwan, Iran, with a six months’ history of left testis painless enlargement without any known predisposing factors. Outpatient examination findings were normal. Family history and past medical history of the patient was unremarkable. General examination was insignificant except for a mass (about 4 cm in greatest diameter) in the left testis. No other masses were detected anywhere else. Routine laboratory investigations were normal. Alpha-fetoprotein (AFP), lactate dehydrogenase (LDH) and Beta-human chorionic gonadotropin (B-HCG) levels in serum were 2.61 ng/mL, 321 IU/L and <0.1 mIU/mL, respectively which were in normal limit for the age and gender. The patient underwent ultrasound imaging, which showed a solid heterogeneous mass in the left testis. The patient was treated by inguinal high ligation radical orchiectomy due to suspicion of testicular malignancy. He passed the procedure without complications. The received specimen for histopathologic assessment consisting of left testis was measured to be 6.5 × 4.3 cm and attached spermatic cord was measured 10 cm of length and 2 cm of diameter. Sections showed an intratesticular whitish lesion with whorling pattern in upper pole measuring 4 × 3 × 3cm (Figure 1). Histological examinations revealed a highly cellular neoplastic areas composed of atypical cells with spindle to oval nuclei and scant cytoplasm which showed marked pleomorphism. Areas of hemorrhage and necrosis were also seen. Mitosis was easily found (Figures 2 & 3). No tumor extension to the spermatic cord or tunica vaginalis was identified. The main differential diagnosis was malignant spindle cell tumor. The specimen was sent to the Sina hospital pathology laboratory in Tehran for supplementary evaluation. Immunohistochemical (IHC) staining showed a positive reactivity for smooth muscle actin (SM-A), H-caldesmon, and Desmin. No staining was observed for cytokeratin (CK), CD-34, CD-117, Discovered on GIST-1 (DOG-1), and SRY-related HMG-box 10 (SOX-10). Proliferative activity (Ki-67) was 30% (Figure 4). So, our case was compatible with low grade leiomyosarcoma and diagnosis was confirmed. After left radical orchiectomy, the patient received no adjuvant therapy because no evidence of invasion to lymph node or other complications were observed. Follow up of the patient after 1 year shows no evidence of recurrence and he is asymptomatic until present.

## Discussion

The reason of primary testis leiomyosarcoma is controversial. Its source has been recognized from smooth muscle cells from tunica propria or tunica albugínea or seminiferous tubules or blood vessel walls (4). Primary leiomyosarcoma in testis was an unusual finding with only some reported cases in published articles (5). Said that, to date, only 15 patient with primary leiomyosarcoma in testis have been reported (3). Reported cases with primary leiomyosarcoma in testis had the average age of 50 years which is compatible with our case. According to reported cases, young patients essentially had risk factors such as anabolic steroid use for long time, other germ cell tumors in testis, chronic inflammation in testis and local radiotherapy for previous leukemia (6). Most reported cases have been in small series in relationship with germ cell tumors (7). Symptoms and signs of these tumor is not different from other testis cancers and the pathological stage classification in patients who were reported in the literature, have constantly been low stage (8). AFP, HCG-β and LDH assessed as tumor markers in recent published studies were in the normal limit (9) which is similar to our case. So, a diagnosis of intra-testicular leiomyosarcoma should only be made after thorough gross and microscopic examinations (10). It is sometime difficult to discriminate intra-testicular leiomyosarcoma from para-testicular leiomyosarcoma and gross examination is helpful. Also, in younger patients, somatic malignancy derived from teratoma should be excluded (11). So, received specimen to our pathology department underwent many sections to rule out teratoma. Close observation after radical orchiectomy could be a good choice of management for testicular leiomyosarcoma, which seemed to have run an indolent course compared to other testicular tumors (12). Chemotherapy regime with adriamycin or adriamycin in addition ifosfamide has been the basis of treatment for invasive or metastatic leiomyosarcoma (5). Retroperitoneal lymph node dissection (RPLND) in leiomyosarcomas may possibly not be required if imaging evaluation exposes no suspected lymph nodes. Thus, RPLND is controversial. There is considerable different approach of cancer management for leiomyosarcoma (13). The prognosis of leiomyosarcoma in testis is difficult to assess; but the increased mitotic rate is considered as a main indication for malignancy potential. Cancers with smooth muscle origin with higher than 5 mitoses for each 10 high power fields are classified as malignant. Folpe and Weiss study showed every mitotic rate in smooth muscle tumor with nuclear pleomorphism must be categorized as indicator for malignant performance. Nevertheless, further knowledge for extraordinary tumors is needed prior to accurate prognosis assessment (14). These tumors have complexity for assessment due to present various differential diagnosis and low incidence.

## Conclusion

There are limited information in the literature about the leiomyosarcoma of testis in terms of nature, histomorphology appearance, prognostic indicators, survival rate, and treatment/management due to its rare occurrence. Previous studies reveal that these tumors may be treated if diagnosed early as well as these tumors need to be differentiated from other conventional tumors of the spermatic cord origin and those related to the germ cell tumors.
